# 

**Published:** 1892-09-03

**Authors:** 


**Notices of Births, Deaths, and Marriages,** are inserted in this column at a charge of 2s. 6d. for an announcement not exceeding four lines.

### NOTICE TO CORRESPONDENTS.

All MS., Letters, Books for Review, and other matters intended for the Editor should be addressed The Editor, The Lodge, Porchester Square, London, W.

The Editor cannot undertake to return rejected M.S., even when accompanied by stamped directed envelope.

**Notice to Advertisers and Subscribers.**

All Advertisements, Orders for Copies of The Hospital, and Business Communications should be addressed to The Publisher, *not to the Editor*, The Hospital, 140, Strand, W.C.

The Cost of Subscription, post free, is as follows:—Per week, 2½d.; per quarter, 2s. 6d.; per half-year, 5s.; per annum, 8s. 6d. Foreign:—Per annum, 10s. 8d.; payable, in all cases, in advance.

**"Burdett's Hospital Annual," 1891—1892.**

Third year of publication. 600 pp. Boards, gilt lettering. A most complete guide to British and Colonial Hospitals, Dispensaries, Nursing and Convalescent Institutions, and Asylums. Price 3s. 6d. Published by The Scientific Press (Limited), 140, Strand, W.C.

**NOTICE.—The Index for Vol. XI. (ending March, 1892) is on sale at the Office of this Journal, price 2½d., post free.**

### Scraps and Gleanings.

Sir Lyon Playfair, who has left Liverpool for America for a three months' tour, will take the title of Baron Playfair.

Her Majesty has been pleased to confer a good service pension of £100 per annum upon Colonel G. H. D. Macpherson, on half-pay.

During the fortnight ending Saturday, the 20th, there were 3,428 patients under treatment in the Metropolitan Board Hospitals; of this number 3,022 were scarlet fever cases.

The number of in-patients admitted to the Lloyd Cottage Hospital, Bridlington, during the past year was 109, and of out-patients 110. Number of visits paid by patients to the dispensary was 1,396.

The concert season at the Royal Victoria Hall, Waterloo Road, will open on October 6th, when Madame Antoinette Sterling will sing. The usual Tuesday evening science lectures will be given during next month.

The most stringent measures are being adopted by the medical officers and inspectors of the Port of London Sanitary Committee to guard against the introduction of cholera into this country by the Russian pauper immigrants.

At a recent meeting of the British Medical Association it was unanimously resolved that ladies should become members of the Association. This will give them the opportunity of attending and speaking at the meetings of the Association.

At the various London hospitals arrangements have been made for the reception of all cases of suspected cholera. At the docks the Poplar and Seamen's Hospitals have isolated a special ward, and this is being done in the majority of cases.

At the Worthing Infirmary 1,935 out-patients and 173 in-patients were treated during the past year. The receipts (excluding £11 8s. returned Income-tax and £46 from new life governors) amounted to £1,209 0s. 4d., and the expenditure to £1,178 12s. 11d.

The number of in-patients admitted to the Royal Cornwall Infirmary during the year has been larger than for the last twenty years. Ninety-four parishes and districts have furnished in and out-patients. There were 118 medical and 240 surgical cases.

The Committee of the Derbyshire Infirmary Saturday Fund had this year a most enjoyable outing to Ashford-in-the-Water. The party numbered about eighty. They travelled via Longstone and Bakewell through some of the prettiest parts of the county.

The Inverness District Board of Lunacy has resolved to erect a sick hospital in connection with the asylum, to accommodate about 150 patients, on the plan of the hospital lately erected at Montrose. This scheme is adopted to relieve the overcrowded state of the institution.

The doctors have successfully performed the operation of transferring new skin to the nose and face of Miss Philbrick where scars had been left, which would have proved a permanent disfigurement. The skin used for this purpose was taken from the arm of Miss Philbrick's brother.

The Committee of the Manchester and Salford Lock Hospital in their report speak of the result of two years' steady work, in which the number of cases treated had been unusually large. The death of Dr. Alfred Blackmore occurred in 1890, and Mr. Alexander Wilson, F.R.C.S.Eng., was appointed to fill his place.

The Prince of Wales, as Grand Master of English Freemasons, intends to move at the next meeting of the United Grand Lodge, on September 7th, a grant of 300 guineas out of the Fund of General Purposes, to the Lord Mayor's Mansion House Fund for the relief of the distress caused by the late fire in St. John's, Newfoundland.

The station master at Frodsham, Cheshire, has recently had his house, booking offices, and general waiting-room moved six feet back without being taken down. Although the mass to be moved weighed quite 400 tons, the work was accomplished without a hitch, save that a chimney-stack, which cracked, had to be taken down.

A lady has presented a cheque for one thousand pounds to Mr. Salmond, the Secretary of the British Home for Incurables, to endow a bed in memory of her father and mother in the new Home at Streatham, the foundation-stone of which was laid by Princess Christian on July 16th, when a cheque for a similar sum and object was handed to her Royal Highness on behalf of two sisters.

The City of Dublin Hospital having proved inadequate to accommodate the numerous applicants, the board have determined to erect a structure which will about double the present accommodation. An excellent design has been selected and estimates made showing that a hospital according to the plans adopted may be constructed at a cost of from £9,000 to £10,000. Lord Pembroke has promised £6,000, and the subscriptions up to June last were close on £2,000.

In order to meet the staff requirements, two extra rooms have been added to the west-end of the Devizes Cottage Hospital. The whole of the drainage of the hospital has been reconstructed, and the ventilation improved. At the annual meeting of the institution the committee drew attention to the falling off in the amounts received from the country parishes during the last year or two, and trusted that one day in each year should be set apart at each place of worship for collections on behalf of the hospital.

### THE STUDENT OF MEDICINE.

[All communications for this Supplement should be addressed to THE EDITOR of THE HOSPITAL, at 140, Strand, with the words, "Students' Column," written in the left hand top corner of the envelope.]

### APPOINTMENTS.

Bower, Augustus E., M.R.C.S. Eng., L.S.A., appointed Medical Officer for the Eastry District, and for the Workhouse of the Eastry Union. Bromhall, Ernest, M.R.C.S.Eng., L.R.C.P.Lond., appointed Assistant House Surgeon to the General Hospital, Birmingham. Chambers, Henry F. T., L.R.C.P., L.R.C.S.Edin., L.F.P.S.Glas., appointed Medical Officer for the Wareham No. 2 District, and for the Workhouse of the Wareham and Purbeck Union. Edie, Robert, M.B., C.M., appointed House Surgeon to the Manchester Royal Eye Hospital. Harcourt, C. Harold, M.R.C.S.Eng., L.R.C.P.Lond., appointed Assistant House Surgeon to the General Hospital, Birmingham. Haring, Nathan C., M.B.Lond., M.R.C.S., appointed Honorary Assistant Physician to the Manchester Hospital for Consumption and Diseases of the Throat. Harman, Albert B., M.R.C.S., L.S.A., appointed Assistant House Surgeon to the Royal South Hants Infirmary, Southampton. Hill, Frederick A., M.D.Brussels, M.R.C.S.Eng., L.R.C.P.Lond., appointed Medical Officer for the Thorncombe District of the Axminster Union. Jones, Hugh R., M.A., M.D., B.C.Cantab., B.Sc.Lond., appointed Honorary Assistant Surgeon to the Liverpool Infirmary for Children. Paling, Albert, M.R.C.S., L.R.C.P., L.S.A., appointed Assistant Resident Medical Officer to the Whitechapel Infirmary. Thorne, Thorne Berthold B., M.R.C.S., L.R.C.P., appointed Assistant House Surgeon to the Royal Portsmouth Hospital. Williams, Egerton H., M.R.C.S., L.R.C.P., appointed Clinical Assistant to the Whitechapel Infirmary. Dalgetty, Arthur B., M.B., C.M., appointed Medical Assistant Officer Dundee Royal Lunatic Asylum.

### VACANCIES.

The following vacancies are announced this week, the amount of salary in each case being given after the name of the institution:

#### Resident Medical Officers.

Berks Asylum, Moulsford, near Wallingford, Assistant Medical Officer, doubly qualified, unmarried, and under 30 years of age, Salary £100 per annum, rising £10 annually to £120, with furnished apartments, board, &c.

City of London Hospital for Diseases of the Chest, Victoria Park, E., House-Physician for six months, commencing October 1st.

Central London Ophthalmic Hospital, House Surgeon.

Royal Surrey County Hospital, House Surgeon—Salary £80 per annum.

Sussex County Hospital, Brighton, House Surgeon, doubly qualified, unmarried, and under 30 years of age—Salary, £120, rising to £140 per annum, with board, &c.

#### Non-Resident Medical Officers.

Buntingford Union, Medical Officer and Public Vaccinator—Salary £80 per annum, exclusive of medical and vaccination fees. Must reside within the district.

Derby Amalgamated Societies' Medical Association, Assistant Surgeon—Salary £160 per annum, with an allowance of £44 for cab hire, and additional fees for midwifery cases—salary to be advanced £10 in six months after appointment, and £10 per annum afterwards until it reaches £250 per annum.

Dunstable Urban Sanitary Authority, Medical Officer—Appointment for one year—Salary 20 guineas.

London County Council, Assistant Medical Officer of Health, not less than 26 or more than 40 years of age—Salary £300 per annum, rising by annual increments of £50 until it reaches £600 a year.

St. Mary's Hospital, Paddington, W., Physician, Accoucheur.

Sunderland Corporation, Medical Officer of Health for the District of the Borough and Port—Salary £500 per annum as Medical Officer of the borough, £20 for the like office of the Port, and £5 as the Public Analyst.

University of Aberdeen Six Examiners for graduation in Medicine—Grant of £30 per annum each.

University of Glasgow, Assistant Examiner in Medicine—Annual fee for Examinership is £30.

### PASS LISTS.

#### University of London.

INTERMEDIATE EXAMINATIONS IN SCIENCE AND PRELIMINARY SCIENTIFIC (M.B.) EXAMINATION, 1892.

EXAMINATIONS FOR HONOURS.

INORGANIC CHEMISTRY.—First Class: J. W. Shepherd, Int. Sc. (disqualified by age for the Exhibition), Anderson's and Veterinary Colleges, Glasgow, and private tuition; B. B. Turner, Int. Sc. (Exhibition), C. Guilds Central and Birkbeck Institution; E. C. Thompson, Int. Sc., Royal College of Science and University Tutorial College. Second Class: H. M. Atkinson, Int. Sc., Craigmore College and University College, Aberystwith; T. F. Rutter, Int. Sc., University College; H. R. Le Sueur, Int. Sc., University College; D. Owen, Int. Sc., University College, Bangor. Third Class: H. Hills, B.A., Int. Sc.,

### THE STUDENT OF MEDICINE—(*continued*).

Bromley School of Science, King's College, and private study; G. W. Jones, Int. Sc., private study and St. Thomas's Charterhouse School of Science, and J. H. Wolfenden, Int. Sc., St. John's School, Failsworth, Royal College of Science, and University Tutorial College, equal; A. O. Allen, B.A., Int. Sc., private study and University Tutorial College; W. H. Barker, Int. Sc., University College, Aberystwith, and J. P. G. Templeman, Int. Sc., King Edward's High Schools, and Mason's College, Birmingham, equal.

Experimental Physics.—First Class: B. B. Turner, Int.Sc. (Neil Arnott Medal), C. Guilds Central and Birkbeck Institutions. Third Class: W. T. Quin, Int.Sc., Caius Cambridge, and High School, Newcastle-under-Lyne; J. W. Shepherd, Int.Sc., Anderson's and Veterinary Colleges, Glasgow, and private tuition; H. Hills, Int.Sc., Bromley School of Science, King's College, and private study; A. E. Thomas, Int.Sc., Merchant Ventnors' School, and University College, Bristol.

Botany.—First Class: Edith Chick, Int.Sc., University College. Second Class: F. G. Peck, Prel.Sci., University College; A. G. Wilson, Prel.Sci., University College; S. Hunt, Prel.Sci., University College; *A. W. Sikes, Prel.Sci., St. Thomas's Hospital, and Birkbeck Institution; T. E. Lones, Int.Sc., private study. Third Class: Katharine R. Jay, Int.Sc., Bedford, College, London; Alice M. Hughes, Int.Sc., University Colleges, Bangor and Aberystwith; H. J. Starling, Prel.Sci., Guy's Hospital; Emily J. Suffield, Int.Sc., King Edward's High School, Birmingham; C. G. Seligmann, Prel.Sci., St. Thomas's Hospital; F. A. St. John, Int.Sc., St. Mary's Hospital; C. Elizabeth Oldaker, Int.Sc., Newnham, Technical School, Leicester, and private tuition; Lilian A. Silcox, Int.Sc., University College, Liverpool.

Zoology.—First Class: *C. Bolton, Prel. Sci. (Exhibition), University College; †J. H. Tallent, Int. Sc., University College, Birkbeck Institute, and private study; *E. H. Schwarz, Prel. Sci., Royal College of Science and private study; J. E. Gray, Int. Sc., University College. Second Class: M. I. Newbigin, Int. Sc., University College, Aberystwith; B. B. Turner, Int. Sc., C. Guilds Central and Birkbeck Institutions. Third Class: C. J. Coleman, Int. Sc., Mason College and private study; *W. J. Harding, Prel. Sci., St. Bartholomew's Hospital; R. P. Williams, Prel. Sci., King's College; R. Evans, Int. Sc., University College, Bangor; T. E. Lones, Int. Sc., private study.

* These candidates have also passed in the Mathematics of the Intermediate Examination in Science, ahd have thus become admissible to the B. Sc. Examination.

† Obtained the number of marks qualifying for the exhibition or prize.

### Edinburgh University.

The following are the official lists of candidates who passed the first examinations at Edinburgh University last month:—

First Professional Examination.—(* With distinction)—C. W. Anderson, D. N. Anderson, Frederick Anderson, J. W. Anderson, John Ballantyne, N. D. Bardswell, J. A. Bayne (M.A.), J. E. H. Bennett, J. B. Blaikie, A. B. Blair, W. H. Borrie, F. J. C. Y. Botha, Edwin Bramwell, A. S. Brass, T. A. Brennan, H. S. Brockway, R. M. Broughton, G. S. Brown, H. G. Brown, W. J. Buchanan (B.A.), D. A. Cameron, H. J. Cardale, A. G. Carment, Scott Carmichael, S. H. Carr, *W. H. Carse, W. W. Chipman (B.A.), *A. W. G. Clark, J. M. Coates, C. B. Crampton, John Crawford, James Davidson, Priyanath Deb, Maurice Dee, Hugh Dewar, Arthur Dickson, J. H. S. B. Douglas, Stanley Ducat, William Duncan, C. W. Eames, J. W. Edwards, A. W. Ellis, John Ellul, William Evans, F. B. Feast, A. M. Fleming, C. C. Forrester, G. O. Franzen, Frederick Gardiner, W. A. Gemmell, R. M. Gibson, John Gilmour, C. J. Gorringe, James Gray, A. Y. Greenwood, J. M. Grieve (M.A.), Norman Gunn, R. M. Hall, S. H. Hall, F. A. Hardy, C. M. Hector, George Henderson, G. P. Henderson, John Henderson, J. J. Hewison (M.A.), W. H. Hill, John Hirst, J. F. Holden, George Holliday, I. D. C. Howden, A. L. Husband, D. H. Hutchinson, Robert Irvine, Walter Jagger, J. M. Jeffrey, Laurence Ker, James Kirk, B. L. L. Learmonth, R. M. Leith, William Lillie, R. C. Loney, J. R. Lord, P. C. Lüttig (B.A.), W. L. Lyall, Angus Macdonald, F. B. Macdonald, W. M. Macdonald, John M'Donald, Stuart M'Donald, J. A. Macfarlane, T. H. Macfie, D. M. Mackay, Charles MacLaurin, John M'Master, D. D. M'Neill, A. D. M'Pherson (M.A.), Colin M'Vicar (M.A.), W. R. Mander, J. L. Marjoribanks, J. S. Martin, Joseph Mason, James Massey, G. H. Masson, F. H. Merry, J. A. Milroy (M.A.), F. J. R. Momplé, W. A. W. Moore, H. M. Morton, S. K. Mullick, T. M. Ness, M. N. Nicolson, Robert Owen, A. J. Park, H. C. Pearson, J. P. Peterson, Charles Porter, A. S. B. Powell, W. H. Price, R. G. Ralston, J. A. Rees, William Ritchie, W. T. Ritchie, W. H. Robb, David Roger, J. E. Rosenstein, D. R. Rowlands, A. H. Rutherford, C. R. Scott, W. A. Skinner, F. O. de Souza, F. S. Stanwell, James Stenhouse, T. H. Stevenson, G. E. Stewart, John Stoddart, W. E. W. Strickland, A. C. Sturrock (M.A.), F. V. Sullivan, F. G. Taylor, H. F. L. Taylor, D. G. M. Teague, F. S. C. Thompson, Robert Thornton, F. W. Twidale, W. A. C. Ussher, J. W. de Vos, Andrew Wallace, Henry Waters, T. E. Watson, David Waterston, James Watt (B.A.), H. G. Waugh, G. A. Welsh, A. E. White, Charles R. White, J. H. White, C. S. Wilkinson, A. E. Williams, H. N. Williams, J. T. Williams, W. O. Williams, W. W. Wood, A. D. Yule.

